# Molecular characterization of *Cryptosporidium* in wild rodents from the Inner Mongolian Autonomous Region and Liaoning Province, China: assessing host specificity and the potential for zoonotic transmission

**DOI:** 10.3389/fvets.2024.1406564

**Published:** 2024-05-30

**Authors:** Li Liu, Qunfang Xu, Aiying Jiang, Fansheng Zeng, Wei Zhao, Feng Tan

**Affiliations:** ^1^Department of Public Health and Laboratory Medicine, Yiyang Medical College, Yiyang, China; ^2^School of Basic Medical Sciences, Wenzhou Medical University, Wenzhou, China

**Keywords:** *Cryptosporidium*, prevalence, wild rodent, genotyping, public health, China

## Abstract

**Introduction:**

Wild rodents are key hosts for *Cryptosporidium* transmission, yet there is a dearth of information regarding their infection status in the Inner Mongolian Autonomous Region and Liaoning Province of China. Therefore, the present study was conducted to determine the prevalence and genetic characteristics of *Cryptosporidium* among wild rodents residing in these two provinces.

**Methods:**

A total of 486 rodents were captured, and fresh feces were collected from each rodent’s intestine for DNA extraction. Species identification of rodents was performed through PCR amplification of the vertebrate cytochrome b (*cytb*) gene. To detect the presence of *Cryptosporidium* in all fecal samples, PCR analysis and sequencing of the partial small subunit of the ribosomal RNA (*rRNA*) gene were performed.

**Results:**

Four species of rodents were identified: *Rattus norvegicus*, *Mus musculus*, *Apodemus agrarius*, and *Cricetulus barabensis*. Positive results for *Cryptosporidium* were obtained for 9.2% (18/195), 6.6% (7/106), 5.6% (5/89), and 6.3% (6/96) of these rodents, respectively, with an average infection rate of 7.4% (36/486). The identification revealed the presence of five *Cryptosporidium* species, *C. ubiquitum* (*n* = 8), *C. occultus* (*n* = 5), *C. muris* (*n* = 2), *C. viatorum* (*n* = 1), and *C. ratti* (*n* = 1), along with two *Cryptosporidium* genotypes: Rat genotype III (*n* = 10) and Rat genotype IV (*n* = 9).

**Discussion:**

Based on the molecular evidence presented, the wild rodents investigated were concurrently infected with zoonotic (*C. muris*, *C. occultus*, *C. ubiquitum* and *C. viatorum*) as well as rodent-adapted (*C. ratti* and Rat genotype III and IV) species/genotypes, actively participating in the transmission of cryptosporidiosis.

## 1 Introduction

*Cryptosporidium*, a parasitic apicomplexan organism, infiltrates the epithelial cells of the small intestine, leading to infections that are the second most prevalent cause of severe diarrhea among young children residing in regions with limited resources (1). Additionally, *Cryptosporidium* is a significant opportunistic pathogen among immunocompromised individuals, such as those living with Human Immunodeficiency Virus (HIV), transplant recipients, cancer patients undergoing chemotherapy, and those undergoing hemodialysis treatment (2). Moreover, water-borne and food-borne outbreaks of *Cryptosporidium* are common among the general population. Globally, more than 1,200 outbreaks have been attributed to the transmission of *Cryptosporidium* through waterborne sources (3). Additionally, over 8 million cases of cryptosporidiosis were reported annually due to foodborne outbreaks (4). Therefore, cryptosporidiosis holds immense significance in public health, necessitating proactive measures to prevent and control its occurrence.

By using genotyping technology, over 170 species and genotypes of *Cryptosporidium* have been identified, existing across a diverse range of hosts (5). Human cryptosporidiosis is primarily attributed to either the anthroponotic *C. hominis* or zoonotic *C. parvum*. Additionally, humans can become infected with another 20 species/genotypes of *Cryptosporidium* (6). Although these infections occur at a lower frequency, recently, there has been a noticeable increase in reports of human infections caused by species other than *C. hominis* and *C. parvum*, such as *C. meleagridis*, *C. ubiquitum*, *C. cuniculus*, *C. andersoni* and *C. viatorum* (5, 6). These species of *Cryptosporidium* possess the ability to infect a diverse array of animals, and the majority of human infections caused by them may occur through animals, either via direct contact or ingestion of feces-contaminated oocysts in water or food (6). To effectively contain the transmission of *Cryptosporidium*, it is crucial to embrace a “One Health” approach that recognizes the intricate interdependence between humans, animals, and the environment (7). Rodents, which are widely distributed globally with a vast array of activities, maintain close ties to humans, animals, and the environment. Consequently, they exert significant influence on their ecosphere, particularly due to their ability to transmit *Cryptosporidium* oocysts into the environment, thereby affecting both humans and animals (8).

Extensive research has been conducted on rodents infected with *Cryptosporidium*, revealing an average prevalence of 19.8% when molecular detection methods are employed (8). Molecular confirmation has identified more than 26 species and 59 genotypes of *Cryptosporidium* across more than 54 rodent species (5, 8). Although most species and genotypes are host-specific or exhibit a limited host range, virtually all known *Cryptosporidium* species and genotypes capable of infecting humans have been detected in rodents (5, 8). Consequently, rodents pose a significant public health risk as reservoirs of zoonotic *Cryptosporidium* species. To effectively evaluate the prevalence of *Cryptosporidium* in rodents and support the development of policies aimed at preventing its transmission to humans and other animals, continuous monitoring of *Cryptosporidium* in rodents, particularly wild rats, is imperative, especially in regions where no sampling conducted before.

In China, the Inner Mongolian Autonomous Region and Liaoning Province are mainly dependent on agriculture and animal husbandry as their economic sources. Rodents are widely distributed in these regions and are active on farms and livestock farms. However, currently, the prevalence of *Cryptosporidium* in rodents, especially wild ones, in these two provinces is still unclear. Therefore, this study aimed to conduct a molecular diagnosis of *Cryptosporidium* in wild rodents in the Inner Mongolian Autonomous Region and Liaoning Province of China, determine *Cryptosporidium* infection rates and evaluate the risk of zoonotic transmission of *Cryptosporidium* at the species level.

## 2 Materials and methods

### 2.1 Ethical concerns

The protocols used in the present study underwent a meticulous review process and were ultimately approved by the Research Ethics Committee of Wenzhou Medical University (approval number SCILLSC-2021-01).

### 2.2 Sample collection

**Figure 1:**
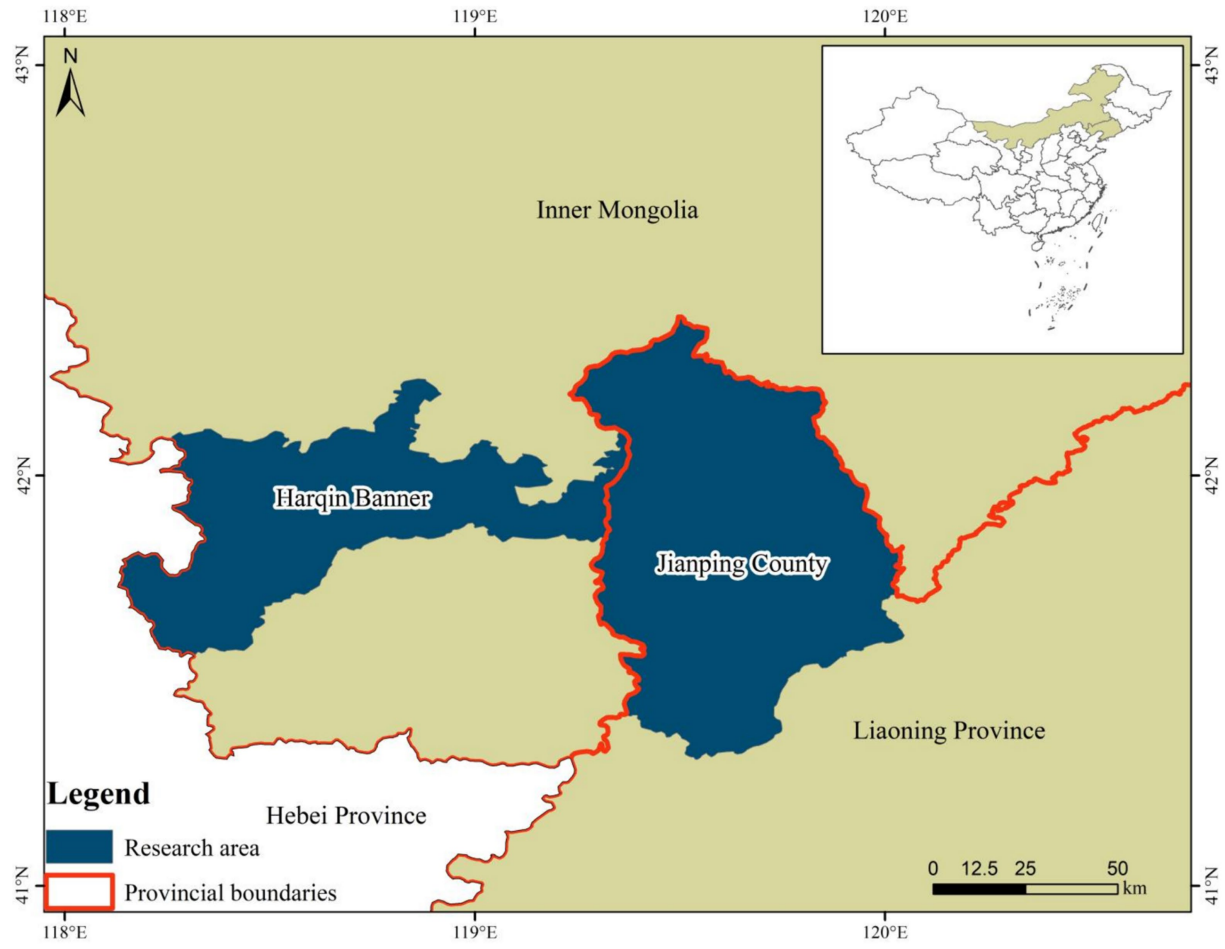
Map of the wild rodent sampling location in the Inner Mongolian Autonomous Region and Liaoning Province, China.

Between November 2023 and February 2024, a cumulative total of 486 wild rodents were collected, with 229 rodents originating from Harqin Banner in Inner Mongolia and 257 rodents originating from Jianping County in Liaoning Province, China (Figure 1 and Table 1). Rodents were captured by utilizing cage traps baited with a mixture of peanut and sunflower seeds. For each designated capture location, approximately 50 cage traps were methodically placed in a straight line, ensuring a uniform spacing of 5 meters between each trap and effectively establishing transects. At 4:00 PM, the transects were positioned and retrieved the following morning at 8:00 AM. Each rodent captured was euthanized humanely via CO_2_ asphyxiation and promptly transported to the laboratory within 48 h, ensuring its safety in sealed containers containing ice. A fecal sample weighing 0.5 grams was collected from the rectum of each rodent.

**Table 1:** Prevalence and distribution of *Cryptosporidium* species/genotypes in the investigated rodents from the Inner Mongolian Autonomous Region and Liaoning Province of China.

Regions	Rodent species	Positive/examined (%)	Species/genotype of *Cryptosporidium* (*n*)
Liaoning (Jianping)	*Apodemus agrarius*	3/62 (4.3)	*C. ubiquitum* (1), *C. viatorum* (1), Rat genotype III (1)
	*Cricetulus barabensis*	1/36 (2.8)	*C. occultus* (1)
	*Mus musculus*	1/28 (3.6)	*C. muris* (1)
	*Rattus norvegicus*	11/103 (10.7)	Rat genotype III (4), Rat genotype IV (4), *C. occultus* (3)
	Subtotal	16/229 (7.0)	Rat genotype III (5), *C. occultus* (4), Rat genotype IV (4), *C. muris* (1), *C. ubiquitum* (1), *C. viatorum* (1)
Inner Mongolia (Harqin Banner)	*Apodemus agrarius*	2/27 (7.4)	Rat genotype III (2)
	*Cricetulus barabensis*	5/60 (8.3)	Rat genotype IV (4), *C. occultus* (1)
	*Mus musculus*	6/78 (7.7)	Rat genotype III (3), *C. ubiquitum* (2), *C. muris* (1)
	*Rattus norvegicus*	7/92 (7.6)	*C. ubiquitum* (5), *C. ratti* (1), Rat genotype IV (1)
	Subtotal	20/257 (7.8)	*C. ubiquitum* (7), Rat genotype IV (5), Rat genotype III (5), *C. muris* (1), *C. occultus* (1), *C. ratti* (1)
Total		36/486 (7.4)	Rat genotype III (10), Rat genotype IV (9), *C. ubiquitum* (8), *C. occultus* (5), *C. muris* (2), *C. viatorum* (1), *C. ratti* (1)

### 2.3 DNA extraction

Exclusively designated for DNA extraction, 0.2 grams of each fecal sample was processed, while the remaining portion was preserved as a backup and stored at a chilled temperature of −80°C. Using the QIAamp DNA Mini Stool Kit (Qiagen, Germany), genomic DNA was extracted from each processed sample. During the extraction process, the lysis temperature was increased to 95°C, while all other steps were performed strictly according to the manufacturer’s guidelines. Subsequently, the DNA was reconstituted in 200 μL of AE elution buffer, provided with the kit, and was subsequently stored at −20°C prior to PCR analysis.

### 2.4 Identification of rodent species

To identify the rodent species, the vertebrate cytochrome b (*cytb*) gene (421 bp) was amplified via PCR from the fecal DNA. The primer sequences were 5’-TACCATGAGGACAAATATCATTCTG-3′ and 5’-CCTCCTAGTTTGTTAGGGATTGATCG-3′, and PCR conditions were as follows: 35 cycles of denaturation at 94°C for 30 s, annealing at 51°C for 30 s, and extension at 72°C for 30 s. Initial denaturation was performed at 94°C for 5 min, followed by a final extension at 72°C for 5 min. These conditions followed the previously described protocol by Verma and Singh (10).

### 2.5 *Cryptosporidium* genotyping

All DNA samples were subjected to nested PCR utilizing primers previously developed by Xiao et al. in 1999 to amplify an 830 bp fragment of the partial small subunit ribosomal RNA (*SSU rRNA*) gene of *Cryptosporidium* (9). The primers used for the primary PCR were 5’-TTCTAGAGCTAATACATGCG-3′ and 5’-CCCTAATCCTTCGAAACAGGA-3′, while the primers used for the secondary PCR were 5′-GGAAGGGTTGTATTTATTAGATAAAG-3′ and 5’-AAGGAGTAAGGAACAACCTCCA-3′. Both PCR amplification steps were conducted under identical conditions, commencing with initial denaturation at 94°C for 3 min. This was followed by 35 cycles of denaturation at 94°C for 45 s, annealing at 55°C for 45 s, extension at 72°C for 1 min, and a final extension at 72°C for 7 min. TaKaRa Taq DNA Polymerase was used for PCR amplification, with positive controls using DNA from chickens infected with *C. baileyi* and negative controls using deionized water without DNA templates. PCR products were analyzed via gel electrophoresis on a 1.5% agarose gel in TAE buffer, with GelRed (Biotium Inc., Fremont, California, United States) serving as the staining agent.

### 2.6 Sequencing analysis

The PCR products of the expected size were purified using a DNA gel purification kit from Sangon Biotech (Shanghai, China). These purified products were then sequenced using the Sanger sequencing method by Sangon Biotech (Shanghai) Co., Ltd., on an ABI Prism 3,730 XL DNA analyzer. Sequencing was performed with the same primers used for the secondary PCR and was facilitated by a BigDyeTerminator v3.1 cycle sequencing kit (Applied Biosystems, Carlsbad, CA, United States). To guarantee the precision of the nucleotide sequence, sequencing was carried out from both ends of the product, and further PCR products were sequenced whenever mutations were identified. After acquiring the sequences, they were carefully edited using DNASTAR Lasergene version 7.1.0 and aligned with reference sequences retrieved from the National Center for Biotechnology Information (NCBI) (https://www.ncbi.nlm.nih.gov/) using the basic local alignment search tool (BLAST) and ClustalX 2.0 software (http://www.clustal.org/) to accurately identify the *Cryptosporidium* species.

### 2.7 Phylogenetic analyses

The SSU rRNA sequences of *Cryptosporidium* spp. obtained in this study were combined with reference sequences to construct a phylogenetic tree using Mega 7.0 software. The Tamura–Nei model-based Maximum Likelihood method was chosen to analyze the phylogenetic relationships. To ensure the reliability of the evolutionary tree, a bootstrap analysis was conducted with 1,000 replicates. The reference sequences necessary for tree construction were retrieved from GenBank and previous research studies.

### 2.8 Statistical analyses

Statistical analysis was performed utilizing SPSS version 22.0 (SPSS Inc., United States). The chi-square test was utilized to determine the disparities in the occurrence of *Cryptosporidium* spp. across diverse regions and rodent species. A *p* value less than 0.05 was considered to indicate statistical significance.

### 2.9 Nucleotide sequence accession numbers

The nucleotide sequences of *Cryptosporidium* obtained in this study have been deposited in the GenBank database under accession numbers PP527771 to PP527783.

## 3 Results

### 3.1 Rat species identification

In this study, PCR and sequencing analysis of the *cytb* gene revealed the presence of four rodent species: *Apodemus agrarius* (*n* = 89), *Cricetulus barabensis* (*n* = 96), *Mus musculus* (*n* = 106) and *Rattus norvegicus* (*n* = 195). No additional data were gathered for these wild rodents (Table 1).

### 3.2 Prevalence of *Cryptosporidium* infection

Nested PCR was performed on 486 fecal samples to assess the presence of *Cryptosporidium* species by analyzing the *SSU rRNA* gene. The results revealed that 36 samples were positive for this parasite, yielding an average infection rate of 7.4%, with 7.0% (16/229) in Liaoning (Jianping) and 7.8% (20/257) in Inner Mongolia (Harqin Banner) (Table 1). Statistical analysis did not indicate any significant differences in *Cryptosporidium* prevalence between the two regions (*χ*^2^ = 0.11, df = 1, *p* = 0.74). Regarding rodent species variation, the highest infection rate of *Cryptosporidium* was observed for *R. norvegicus* (9.2%; 18/195), followed by *M. musculus* (6.6%; 7/106), *A. agrarius* (5.6%; 5/89), and *C. barabensis* (6.3%; 6/96). The difference in the infection rate of *Cryptosporidium* among the rodent species groups was not statistically significant (*χ*^2^ = 1.65, df = 3, *p* = 0.65).

### 3.3 Distribution of *Cryptosporidium* species/genotypes

Five species of *Cryptosporidium*, namely, *C. ubiquitum* (*n* = 8), *C. occultus* (*n* = 5), *C. muris* (*n* = 2), *C. viatorum* (*n* = 1), and *C. ratti* (*n* = 1), as well as two genotypes—*Cryptosporidium* Rat genotype III (*n* = 10) and *Cryptosporidium* Rat genotype IV (*n* = 9)—have been identified through sequencing the PCR products of 36 *Cryptosporidium*-positive samples (Table 1).

In Liaoning, *Cryptosporidium* Rat genotype III emerged as the dominant species, accounted for 31.3% (5/16) of the positive samples, followed by *C. occultus* and *Cryptosporidium* Rat genotype IV each comprising 25.0% (4/16). The remaining three species, *C. muris*, *C. ubiquitum*, and *C. viatorum*, contributed equally with a share of 3.3% (1/16) each. On the other hand, in Inner Mongolia, *C. ubiquitum* emerged as the predominant species, accounting for 35.0% (7/20) of the positive samples. *Cryptosporidium* Rat genotype III and *Cryptosporidium* Rat genotype IV followed closely, each comprising 25.0% (5/20) of the positive samples. The remaining species, *C. muris*, *C. occultus*, and *C. ratti*, contributed 5.0% (1/20) each (Table 1).

Among the different rodent species, *R. norvegicus* carried a diverse range of species/genotypes of *Cryptosporidium*, including *C. ubiquitum*, *C. ratti*, *C. occultus*, *Cryptosporidium* Rat genotype III and *Cryptosporidium* Rat genotype IV. In contrast, *C. barabensis* was limited to carrying only *C. occultus* and *Cryptosporidium* Rat genotype IV. The remaining two rodent species each harbored three species/genotypes: *C. ubiquitum*, *C. viatorum* and *Cryptosporidium* Rat genotype III in *A. agrarius* and *C. ubiquitum*, *C. muris* and *Cryptosporidium* Rat genotype III in *M. musculus* (Table 1).

### 3.4 Genetic identification of *Cryptosporidium* species/genotypes

Among the nine SSU rRNA sequences belonging to *Cryptosporidium* Rat genotype IV, six sequences were found to be identical to each other, sharing perfect 100% similarity with the *Cryptosporidium* genotype W19 variant sequence (AY737581) previously isolated from water samples in the United States (US). The three remaining sequences of *Cryptosporidium* rat genotype IV were identical to each other and had not been previously described. They exhibited a remarkable similarity of 99.87% to the *Cryptosporidium* genotype W19 variant sequence (AY737582), which was also detected in US waters. The sole difference among them was a single nucleotide substitution, specifically from A to G, at position 441 (Table 2).

**Table 2:** Sequence similarity analysis of *Cryptosporidium* species/genotypes in this study with reference sequences from GenBank.

*Cryptosporidium* species/genotypes (n)	Accession number	Identities (Nucleotide difference at position)	Ref accession numbers in host from country
*C. muris* (2)	PP527778	100% (/)	MW090931 in wastewater and sewage from China
*C. occultus* (2)	PP527779	100% (/)	MG699179 in *M. unguiculatus* from the Czech Republic
*C. occultus* (3)	PP527780	99.51% (T to A at position 444, a T insertion at position 446, A to T at positions 482 and 488)	MG699179 in *M. unguiculatus* from the Czech Republic
C. ratti (1)	PP527782	99.87% (G to T, at position 81)	MT504541 in *R. norvegicus* from the Czech Republic
C. ubiquitum (8)	PP527777	100% (/)	MW043441 in cattle from Bangladesh
C. viatorum (1)	PP527783	99.87% (G to A, at position 502)	MK522269 in *Leopoldamys edwardsi* from China
Rat genotype III (2)	PP527781	99.01% (12 nucleotide differences, including 9 substitutions and 3 insertions)	JX294363 in wild black rats from Australia
Rat genotype III (5)	PP527773	100% (/)	JX294367 in wild black rats from northern Australia
Rat genotype III (1)	PP527774	99.51% (T to C at position 482 and T delete at positions 439 and 440)	JX294367 in wild black rats from northern Australia
Rat genotype III (1)	PP527775	99.15% (T to G at position 93, G to A at position 398, T to C at position 438, T delete at positions 439 and 440, T to C at position 481, G to A at position 599)	JX294367 in wild black rats from northern Australia
Rat genotype III (1)	PP527776	99.27% (T to C at position 481, G to A at position 559, T to C at position 593, T to A at position 683, G to A at position 739, and G to T at position 755)	JX294367 in wild black rats from northern Australia
Rat genotype IV (6)	PP527771	100% (/)	AY737581 in water from the US
Rat genotype IV (3)	PP527772	99.87% (A to G, at position 441)	AY737582 in water from the US

Ten SSU rRNA sequences of *Cryptosporidium* Rat genotype III revealed five types, with one type represented in five samples sharing an identical sequence (JX294367) with *Cryptosporidium* Rat genotype III from wild black rats in northern Australia. The second type represented two samples were novel, exhibiting 99.01% similarity (12 nucleotide differences, including 9 substitutions and 3 insertions) with JX294363, which was found in wild black rats from Australia. The remaining three types were each found in a single sample and were previously undescribed, differing from the *Cryptosporidium* Rat genotype III sequence (JX294367) of wild black rats from northern Australia by three (T to C at position 482 and T delete at positions 439 and 440), seven (T to G at position 93, G to A at position 398, T to C at position 438, T delete at positions 439 and 440, T to C at position 481, G to A at position 599), and six (T to C at position 481, G to A at position 559, T to C at position 593, T to A at position 683, G to A at position 739, and G to T at position 755) nucleotides (Table 2).

The present study identified eight sequences of *C. ubiquitum* that were consistent with each other and exhibited 100% similarity to the *C. ubiquitum* sequence (MW043441) isolated from cattle in Bangladesh. The two *C. muris* sequences were also identical and exhibited 100% similarity with MW090931, which was isolated from wastewater and sewage in Guangzhou, China (Table 2).

Among the five sequences of *C. occultus* obtained in this study, two exhibited 100% homology with MG699179, which was identified in *Meriones unguiculatus* from the Czech Republic. The remaining three sequences of *C. occultus* were homologous to each other and were novel, sharing 99.51% similarity with MG699179, differing by four nucleotides (Table 2).

The sequences of *C. ratti* and *C. viatorum* identified here were novel and differed by one nucleotide from MT504541 in *R. norvegicus* in the Czech Republic and from MK522269, which was found in *Leopoldamys edwardsi* from China (Table 2).

**Figure 2:**
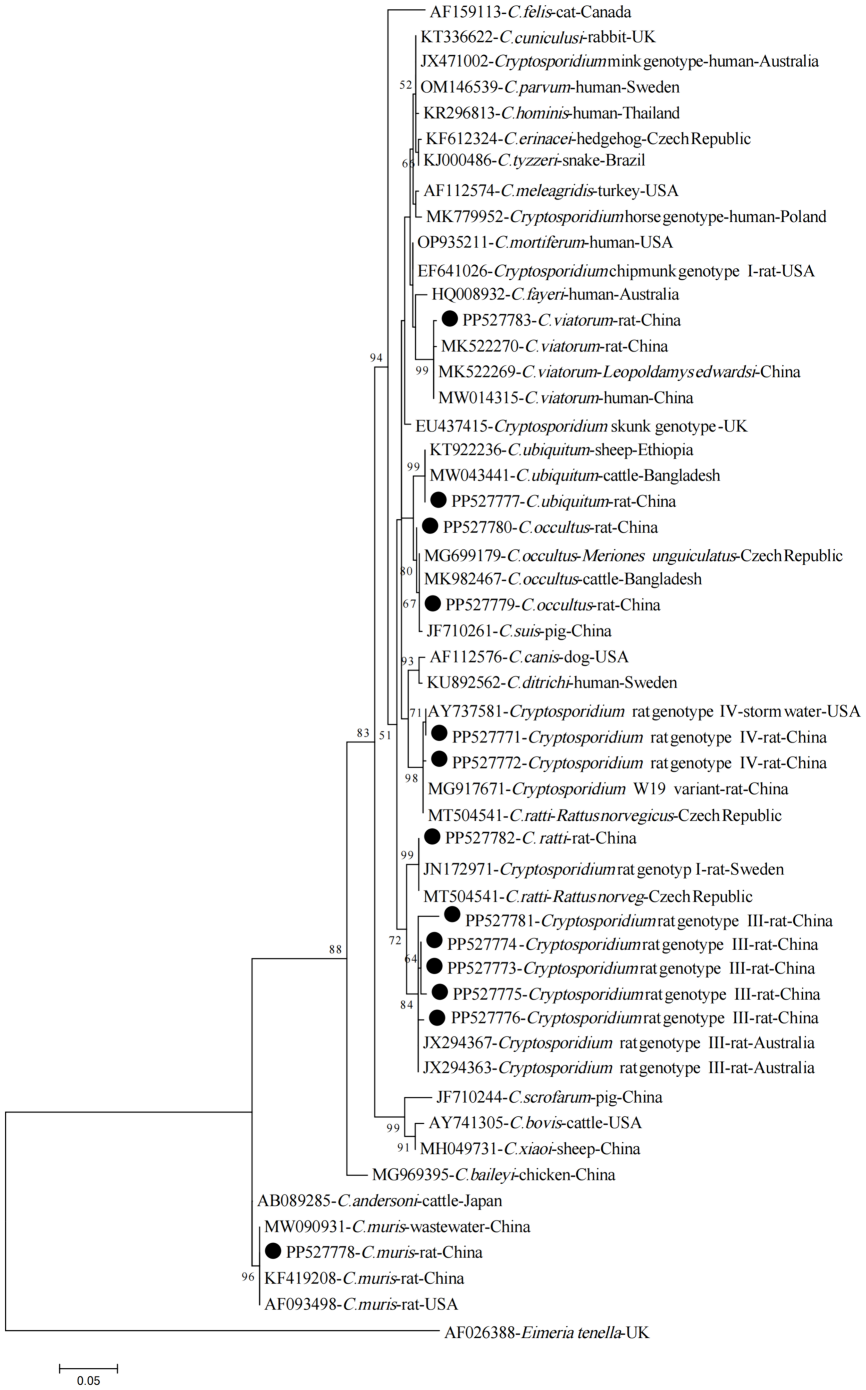
Phylogenetic tree of *Cryptosporidium* species based on partial *SSU rRNA* gene sequences (~800 bp). The tree was generated using the Maximum Likelihood method based on the Tamura-Nei model. Bootstrap values (> 50%) derived from 1,000 replicates are displayed to the left of the nodes for reliability assessment. The sequences generated in the present study are indicated with solid circles.

The phylogenetic analysis of the ssu rRNA sequences has confirmed that the sequences obtained in the present study, corresponding to *C. viatorum*, *C. ubiquitum*, *C. occultus*, *Cryptosporidium* Rat genotype IV, *C. ratti*, *Cryptosporidium* Rat genotype III, and *C. muris*, have clustered together with their respective reference sequences, forming distinct and clearly identifiable groups within the phylogenetic tree (Figure 2).

## 4 Discussion

*Cryptosporidium* infections among rodents have been reported in 19 countries, with global prevalence rates ranging from 0.7 to 100%. The overall average infection rate for rodents is 19.8%, indicating a widespread distribution of this parasite in rodent populations worldwide (8, 11). The present study revealed an average positive rate of 7.4% (36/486) for *Cryptosporidium* among the surveyed wild rodents. Explaining the disparities in prevalence rates among studies is challenging due to the numerous influencing factors. Although the four wild rodent species (*R. norvegicus*, *M. musculus*, *A. agrarius*, and *C. barabensis*) investigated in this study did not show significant differences in infection rates, rodent species may still have an impact on *Cryptosporidium* infection rates. For instance, Zhang et al. recently summarized the occurrences of *Cryptosporidium* infections across 54 rodent species, encompassing wild, domestic pet, farm, and laboratory animals. Specifically, the prevalence rates among these rodent categories are as follows: 20.5% for wild animals, 27.0% for domestic pets, 14.5% for farm animals, and 2.7% for laboratory animals (8). Additionally, geographical location is another crucial factor, with overall infection rates varying across Asia, Europe, South America, North America, and Africa at 18.6, 28.0, 15.2, 7.3, and 2.2%, respectively (11). However, it is worth noting that these rates could be influenced by the limited number of studies conducted in each region. Specifically, Africa, South America, and North America have only one or two studies, thus limiting their representativeness (8, 11). Therefore, to gain a comprehensive understanding of the epidemiology of *Cryptosporidium* in rodents, it is imperative to conduct broader geographical surveys that encompass a more diverse range of species and individuals.

The present study identified five species and two genotypes of *Cryptosporidium* among the surveyed wild rodents. Among these, *C. ratti* and *Cryptosporidium* Rat genotypes III, and IV are predominantly found in rodents, exhibiting a narrow host range that is typically rodent-specific (5). Although sporadically reported in other animals, such as camels, goats, black bears and cats, these species and genotypes have not been documented in humans and are rarely encountered in other hosts, rendering their potential pathogenicity uncertain (5, 12–15). Nevertheless, their ability to be detected in streams in the US and in raw sewage water in various countries underscores the need for further investigations to delineate their actual host range and assess their impact on public health (16–19).

*Cryptosporidium muris*, a dominant parasite in rodents, has been identified in more than 20 rodent species as well as in pigs, pigeons, camels, black-boned goats, sheep, horses, and captive zoo animals (5, 6). Multiple reports exist of *C. muris* infections in humans, primarily in low-income countries and HIV^+^ patients, with limited reports in high-income nations (20). Although our study identified *C. muris* in only two specimens of *Mus musculus*, this finding not only confirms that *Mus musculus* is the primary host of *C. muris* but also suggests that *C. muris* infection may serve as a significant link in disease transmission to humans and other animals. Additionally, *C. occultus* primarily infects rats and has also been reported in ruminants such as cattle, water buffaloes, yaks, deer, alpacas and bactrian camels (5, 21). Limited human cases have also been reported (22). This study is the first to identify *C. occultus* in *R. norvegicus* and *C. barabensis*, further expanding its host range and indicating that these two rodent species play active roles in the transmission of this parasite.

*Cryptosporidium ubiquitum* and *C. viatorum* are two commonly encountered zoonotic species that infect humans (6). Among these, *C. ubiquitum* often occurs in rodent species, encompassing 21 distinct genotypes, exhibiting an exceptional capacity to infect a diverse array of hosts, such as primates, carnivores, and ruminants (5, 23). In the present study, *C. ubiquitum* was identified in *R. norvegicus*, *A. agrarius*, and *M. musculus*, with a preponderance in *R. norvegicus* from Inner Mongolia. This observation might suggest the potential for cross-species transmission of *C. ubiquitum* between rodents and goats/sheep, considering its widespread detection in these animals in Inner Mongolia (24). Although no human cases of *C. ubiquitum* infection have been documented in this region thus far, the known pathogenicity of this species toward humans cannot be discounted, given its numerous reported cases in the United States, Canada, and the United Kingdom (23). Therefore, individuals, particularly those residing in Inner Mongolia, should exercise caution and refrain from contact with brown rats and other wild rodents to mitigate the risk of cryptosporidiosis transmission from rodent sources. Moreover, it has been confirmed here that *A. agrarius* has been infected with *C. viatorum*, which initially identified in humans in 2012 (25). Cases of *C. viatorum* infection have been documented in 13 countries, including China (22). Currently, *C. viatorum* has only been described in rodent species such as *R. rattus*, *R. lutreolus*, *Leopoldamys edwardsi*, and *Berylms bowersi* (26–28). These findings suggest that rodents serve as the primary hosts for this parasite, further emphasizing their crucial role in its transmission.

## 5 Conclusion

This study revealed a 7.4% infection rate of wild rodents with *Cryptosporidium* spp. in the Inner Mongolian Autonomous Region and Liaoning Province, China. Molecular analysis revealed the presence of nonhuman infectious *C. ratti*, *Cryptosporidium* rat genotypes III and IV, and zoonotic species such as *C. ubiquitum*, *C. occultus*, *C. muris*, and *C. viatorum*. These findings indicate that rodents may play a crucial role in maintaining and disseminating these infections, posing a potential risk to public health. Therefore, a comprehensive multidisciplinary “One Health” approach is imperative to gain a thorough understanding of rodent-related *Cryptosporidium* and potential transmission routes.

## Data availability statement

The datasets presented in this study can be found in online repositories. The names of the repository/repositories and accession number(s) can be found in the article/supplementary material.

## Ethics statement

The animal study was approved by the protocols of the present study underwent a rigorous review process and were ultimately approved by the Research Ethics Committee of Wenzhou Medical University, with the approval number SCILLSC-2021-01. The study was conducted in accordance with the local legislation and institutional requirements.

## Author contributions

LL: Writing – review & editing, Conceptualization, Data curation, Investigation, Methodology, Writing – original draft. Q-X: Methodology, Writing – original draft, Writing – review & editing, Funding acquisition, Resources. AJ: Writing – original draft, Writing – review & editing, Investigation, Methodology. FZ: Investigation, Methodology, Writing – review & editing, Writing – original draft. WZ: Formal analysis, Writing – original draft, Writing – review & editing, Funding acquisition, Resources. FT: Conceptualization, Data curation, Supervision, Writing – original draft, Writing – review & editing.
